# Something of the Tact and Patience Necessary in the Prognosis and Treatment of Chronic Deafness

**Published:** 1899-08

**Authors:** Arthur G. Hobbs

**Affiliations:** Atlanta, Ga.


### SOMETHING OF THE TACT AND PATIENCE NECESSARY IN THE PROGNOSIS AND TREATMENT OF CHRONIC DEAFNESS.*

BY ARTHUR G. HOBBS, M.D.,
ATLANTA, GA.

Walter Besant has defined a "knack" as a something that cannot be taught but must be caught on to. In the prognosis and treatment of chronic deafness "tact" is not unlike this "knack" as defined by the novelist. Here, tact must be caught on to, and it must always follow experience, however naturally it may seem to some in applying it elsewhere without experience.

*Read before Georgia State Medical Association in April and before the Atlanta Society of Medicine in June, 1899.

Patience, like tact, may be born in one: it may be a natural attribute of a hopeful nature or a phlegmatic temperament, but it requires a special nursing and a careful development before its possessor can hope to reach any decided success in the treatment of deafness. It is true that it may be said that such attributes are necessary to success in any branch of medicine or surgery, but the only excuse for the caption of this paper is that, *ceteris parabus*, it is particularly essential here, possibly even more essential than a knowledge of *all* the technique of the conventional methods of treating chronic ear diseases.

Tact will compromise at first on the second best means when the patient seems averse to the first, and thereby soon gain his hearty co-operation, which is so essential. And the exercise of patience will, in the end, gain his intelligent assistance in the application, if need be, of the more effective though more painful methods. Tact will suggest *when* to test the hearing distance; whether, for example, in a quiet moment or during rumbling noises in the streets, and thereby encourage or avoid the natural question as to why the hearing is best, in case it is so, in a railroad train, and at the same time it will greatly assist the examiner in arriving at some form of treatment and consequently at a prognosis. Perhaps better than judgment, tact will decide whether the watch, the clock, the voice, or which of the many testing instruments is best to be used in each case; and to gain much value from the tuning-fork, patience is necessary in teaching the subject what is expected of him and of the test, particularly when it is found advisable to use tuning-forks of different vibrations.

The intricacy and delicacy of the hearing apparatus when regarded from a physiological standpoint presupposes the probability of its many and varied forms of pathology, some of which are masked and not easily recognized; hence the prognosis should always be guarded and often not attempted at the first examination, and indeed, not until after some test treatments have been made tactfully and patiently.

The science of medicine does not always receive its deserts from its votaries; and much more is it true that the art of medicine, which is its practice, is not honored as it should be by the persistent application of tact and patience, which are requisite to any degree of success in other arts even when all the other necessary qualifications exist.

We cannot always comprehend the effects of any one of the many means or methods that may be resorted to in the treatment of nonsuppurative catarrhal deafness otherwise than by patient and tactful trials; and this is none the less true in the diagnosing and in the prognosing of this class of ear diseases.

Since the nicety of distinction as to the cause, the character and the situation of the particular fault in the mechanism of the organ is of more importance than a technical knowledge of the methods, it also requires no little patience and perhaps more tact to make the subject realize that more than one examination is often necessary in order to determine upon a plan of treatment, if any, as well as to arrive at an intelligent answer to his eager questions about the probable results to his hearing, the time required, etc. Such answers can only be given with any degree of certainty after persistent and even painful examinations and tests. The impatient subject rarely considers that we must first find the cause and carefully consider all of its bearings if we would reasonably assure ourselves whether another method of treatment would be effective before a prognosis is possible.

'Tis not the new case of deafness, 'tis not at the time when pain is the most pronounced symptom, 'tis not when the patient is most solicitous about his beginning failure to hear; nor is it in the old cases of necrosis or of exostosis or of ossicular anchylosis, that neither require, or will even admit of any decided operative procedures; 'tis not these that call for so much tact and delicacy, but it is one of those progressive, dry cases of chronic catarrh that has reached a high degree of deafness so slowly and so insidiously as to have been barely perceptible. It is one of those cases that has gone the rounds from one aurist to another, expecting each time some miraculous results in a week, at the hands of him who applies only the old conventional text-book methods. It is one of those cases where the family physician has so long advised the do-nothing plan or has told the parents to let the child's ears alone—that he will outgrow it, when the outgrowing process is usually reversed—the disease grows the fastest. It is one of those cases where the subject lacks persistence or is chronically pessimistic. Indeed, it is in such cases as these that tact and delicacy alone can succeed.

By a rather peculiar coincidence, just at this stage in the preparation of this paper, I received a reprint from an article on "Modern Possibilities in the Treatment of Chronic Catarrhal Deafness," by Dr. Snow, of Syracuse, N. Y., in which his refrain is *persistence* as the price of success. If he would or has already included tact and patience in the one word *persistence* he might well make this word his *shibboleth*, and no one could be surprised at the large percentage of success he reports.

If we should follow the text-books now—the majority of them—and attempt to carry out their too often perfunctory instructions, we would not have the opportunity to daily inflate the Eustachian tubes (and do nothing more) by Politzerization, or with the catheter for six weeks, for the very good reason that, in this day and age, our patients, at least those who are susceptible from a psychological standpoint, would not be with us so long.

The older text-books on the ear seem to have fixed just six weeks as the limit of all human endeavor, and after thirty-six days (or forty-two days if Sundays are not observed) of inflating the tubes (effectively possibly, ineffectively probably), they say no patient need again apply. The authors of most of the text-books, even those whose first or last editions are dated as late as 1898, do not seem to realize, as we may venture to predict they will in their future editions, that most ear diseases primarily have their origin in the throat or post-nasal spaces, and must be treated from that general etiological standpoint.

Ten years ago such a statement as this would have been regarded as rank heresy by the aurist, who even at that recent date chose to so call himself, before he began to learn, but still reluctantly to acknowledge that the open sesame, pathologically speaking, to the ear is through the naso-pharyngeal route. The aurist of to-day is neither tactful nor can he be successful if he is not first a rhinopharyngologist. Such subjects would soon lose their patience with any aurist, and justly so, who did not treat them from this point of view. It must be remembered that I am speaking particularly of the non-proliferative form of deafness. Is it not worth a much longer application than the conventional six weeks of patience and persistence, to gain relief from that hammering and nagging condition, known as tinnitus aurium, which is one of the most constant symptoms of this form of deafness? The causes are numerous; it may be due to an aural polypus situated in the canal and growing from a granulation, or a furuncle; a necrosis either external to the membrane, or as is most probable, having its origin in the middle ear, through a perforation in the M. T. It may be a protrusion of the hypertropied wall of the tympanum; a necrosis of the ossicles; a cicatrix from an old inflammation; an anchylosis; a thickening or a stenosis of the Eustachian canal. Again, the cause may be nasal polypi; post-nasal adenoids situated in the region of the Eustachian opening; an hypertrophied Luschka's tonsil, as well as the pressure of enlarged faucial tonsils. Or indeed the cause may be any form of nasal stenosis that produces mouth-breathing, especially when situated near the fossæ of Rosenmüller. Some of these lesions with their numerous sequelæ may cause only unilateral deafness, yet some of these may require much delicacy in the selection of the means to the end. But it *is* that most lesionless condition, generally known as non-suppurative or non-proliferative chronic catarrh of the middle ear, to which this paper more particularly refers, and it is the one, too, that is calculated to place the aurist at his wits' end and in some cases make him wish that he had never been born, or at least that he had not been born an aurist. It is in just such cases as these that the tactful and persistent adaptation of common sense methods succeed.

Nowhere else is the potency of electricity so exemplified; but this presupposes, of course, a general knowledge of the many varieties of this subtle current in order to make a proper selection of the kind, the character, the amount, the direction of the current, and how to apply it, as well as the duration of the séance. This last perhaps requires the most tact, as the effects of electricity vary so greatly in different patients, and often in the same patient, that there can be no set rule. Indeed, the desired effect may be reached and overdone, or it may not be reached at all, within a certain number of minutes.

It requires more knack than tact perhaps to properly introduce a bougie into the Eustachian tube, but it requires more tact than knack to repeat the operation many times in the same case, since it is the patient who must exercise the patience here; as also in the introduction of liquids or gases into the tube, much delicacy is necessary in order to encourage the patient to exercise sufficient persistence for a second operation.

Can we not then say in conclusion, that in the treatment of chronic deafness, tact is the greatest common divisor and patience the least common denominator?

